# AlphaFold-guided structural analyses of nucleosome binding proteins

**DOI:** 10.1093/nar/gkaf735

**Published:** 2025-08-06

**Authors:** Xin Yang, Haoqiang Zhu, Liuxin Shi, Tingrui Song, Weibin Gong, Shunmin He, Shan Shan, Chunfu Xu, Zheng Zhou

**Affiliations:** Key Laboratory of Epigenetic Regulation and Intervention, Institute of Biophysics, Chinese Academy of Sciences, Beijing 100101, China; National Laboratory of Biomacromolecules, CAS Center for Excellence in Biomacromolecules, Institute of Biophysics, Chinese Academy of Sciences, Beijing 100101, China; College of Life Sciences, University of Chinese Academy of Sciences, Beijing 100049, China; Key Laboratory of Epigenetic Regulation and Intervention, Institute of Biophysics, Chinese Academy of Sciences, Beijing 100101, China; National Laboratory of Biomacromolecules, CAS Center for Excellence in Biomacromolecules, Institute of Biophysics, Chinese Academy of Sciences, Beijing 100101, China; College of Life Sciences, University of Chinese Academy of Sciences, Beijing 100049, China; Key Laboratory of Epigenetic Regulation and Intervention, Institute of Biophysics, Chinese Academy of Sciences, Beijing 100101, China; College of Life Sciences, University of Chinese Academy of Sciences, Beijing 100049, China; Key Laboratory of Epigenetic Regulation and Intervention, Institute of Biophysics, Chinese Academy of Sciences, Beijing 100101, China; College of Life Sciences, University of Chinese Academy of Sciences, Beijing 100049, China; National Laboratory of Biomacromolecules, CAS Center for Excellence in Biomacromolecules, Institute of Biophysics, Chinese Academy of Sciences, Beijing 100101, China; College of Life Sciences, University of Chinese Academy of Sciences, Beijing 100049, China; Key Laboratory of Epigenetic Regulation and Intervention, Institute of Biophysics, Chinese Academy of Sciences, Beijing 100101, China; College of Life Sciences, University of Chinese Academy of Sciences, Beijing 100049, China; Key Laboratory of Epigenetic Regulation and Intervention, Institute of Biophysics, Chinese Academy of Sciences, Beijing 100101, China; National Laboratory of Biomacromolecules, CAS Center for Excellence in Biomacromolecules, Institute of Biophysics, Chinese Academy of Sciences, Beijing 100101, China; College of Life Sciences, University of Chinese Academy of Sciences, Beijing 100049, China; National Institute of Biological Sciences, Beijing 102206, China; Tsinghua Institute of Multidisciplinary Biomedical Research, Tsinghua University, Beijing 102206, China; Key Laboratory of Epigenetic Regulation and Intervention, Institute of Biophysics, Chinese Academy of Sciences, Beijing 100101, China; National Laboratory of Biomacromolecules, CAS Center for Excellence in Biomacromolecules, Institute of Biophysics, Chinese Academy of Sciences, Beijing 100101, China; College of Life Sciences, University of Chinese Academy of Sciences, Beijing 100049, China

## Abstract

The nucleosome, as the fundamental unit of chromatin, interacts with a diverse range of proteins, crucially regulating gene expression. In this study, we introduce an AlphaFold-based algorithm designed to analyze nucleosome-binding proteins from a dataset of over 7600 human nuclear proteins. Using proteins that interact with the nucleosome acidic patch as a benchmark, our screening achieves a successful prediction rate of 77% (23 out of 30 proteins). This predictive approach has led to the identification of ARID4A and ARID4B as novel nucleosome-binding proteins. Additionally, this analytical method was used to study RING-family ubiquitin E3 ligase RNF168, demonstrating that RNF168 dimerization enhances its binding to the nucleosome, a finding confirmed by cryogenic-electron microscopy structural analysis. Our findings offer a rapid and effective method for the discovery and characterization of nucleosome-binding proteins and emphasize the significant role of ubiquitin E3 ligase dimerization in epigenetic regulation.

## Graphical Abstract

**Graphical Abstract:**
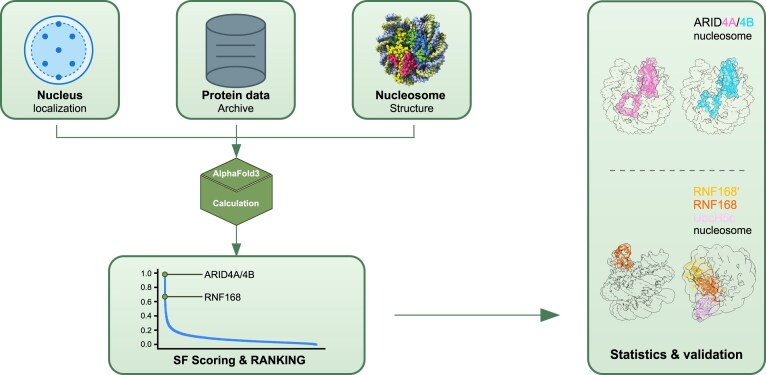

## Introduction

Chromatin, essential to gene expression regulation and genomic stability preservation, comprises DNA and associated histone proteins. The nucleosome core particle serves as the primary structural unit of chromatin and consists of ∼147 base pairs of DNA wrapping around a histone octamer composed of two copies each of histones H2A, H2B, H3, and H4 [1]. This arrangement allows nucleosome to act as a loading platform for DNA and histone modifiers, chromatin remodeling complexes, and epigenetic regulation factors. The interplay between nucleosome and the nonhistone proteins not only compacts DNA to fit within the nucleus but also plays a pivotal role in controlling access to genetic information during transcription, replication, and DNA repair processes [2–5]. It is essential to discover novel nucleosome-binding proteins and elucidate the molecular basis of their nucleosome interaction.

Over the years, structural studies of nucleosome complexes have broadened our understanding of chromatin function [6–8]. High-resolution crystallography and advanced cryogenic-electron microscopy (cryo-EM) have unveiled detailed interactions between DNA and histone proteins, shedding light on how these interactions influenced nucleosome composition [9, 10], nucleosome modifications [11–14], and chromatin remodeling [15–17]. Among all binding hot spots [18] on the nucleosome disk surface, nucleosome acidic patch emerges as a primary docking platform recognized by wide array of protein complexes, including histone chaperones [19–21], chromatin remodelers [17, 22–24], and histone ubiquitination ligases [25–28]. This insight into the acidic patch’s role underscores its value in identifying and evaluating potential nucleosome binders.

Despite the progress, the study of nucleosome complexes faces challenges due to the high costs, time demands, and complex composition of nucleosome-binding proteins, which slow the progress in understanding chromatin mechanics and functions. The emergence of highly accurate protein structure prediction tools like AlphaFold presents an unprecedented opportunity to revolutionize research by enabling hypothesis-driven structural predictions [29–31]. This approach allows for systematic exploration and prediction of the functions of nucleosome variants and their binders, although challenges remain, particularly in predicting complex multi-component assemblies [31–33]. Despite the remarkable advancements, challenges remain in predicting structures affected by chirality errors, chain overlap, conformational dynamics, and most critically, the multi-component complex with heterogeneous assemblies, such as histone ubiquitination ligases [31, 34].

In this study, we reported an AlphaFold-based strategy to predict nucleosome binding proteins from a dataset of over 7600 human nuclear proteins. Our *in silico* screening has efficiently identified novel nucleosome binders and highlighted the significant role of RNF168 dimerization in enhancing nucleosome binding. Integrating these findings with studies of biological interaction networks could accelerate the discovery of new chromatin functions and enrich our understanding of epigenetic regulation.

## Materials and methods

### Proteome acquisition

At the FTP sites of UniProt database, we first navigated to download human reference proteomes (one protein sequence per gene version) in FASTA file with 20 654 proteins. Then performed GO enrichment analysis of cellular components by annotation tool on the QuickGO website. The parameters involved in GO analysis are selected “9606 Homo sapiens” in Taxon and selected “GO:0005634″ in GO terms before exporting 100 220 annotations to a Tab-delimited (TSV) file. Next, we obtained the intersecting set of UniProt IDs shared by the files, resulting in a collection of nuclear proteins (7655) that met the following process criteria.

### Preparation of data for web server prediction

**Figure 1.**
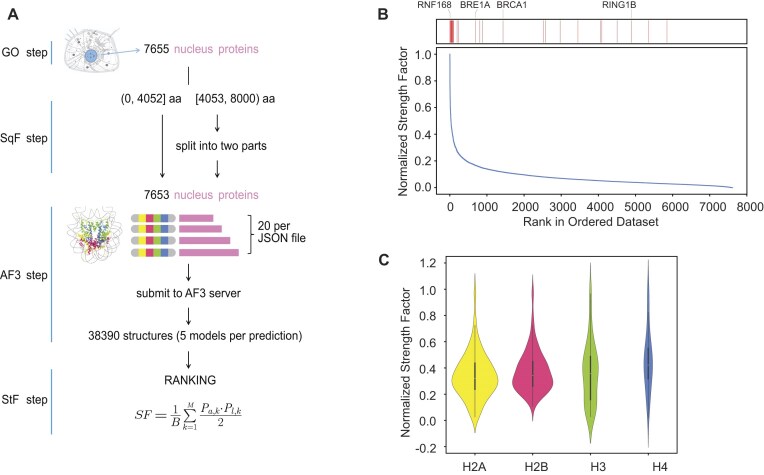
Workflow and results of AlphaFold3-guided nucleosome binding protein prediction. (**A**) Schematic of the AF3-based computational approach for predicting nucleosome binding proteins. The diagram shows the selection of human nuclear proteins (GO step), sequence filtering (SqF step), submission of their sequences to AF3 (AF3 step), and predicted structure filtering (StF step). From an initial set of 7655 nuclear proteins, two exceeding 8000 residues were excluded. The remaining 7653 proteins were processed as follows: 25 proteins exceeded the predictor’s length limit (4052 residues) and were each split into two segments. Each segment underwent prediction, generating five structural modes per segment (25 proteins × 2 segments × 5 modes = 250 predictions). The remaining 7628 proteins fell within the length limit and each generated five structural modes (7628 proteins × 5 modes = 38 140 predictions). This yielded a total of 38 390 predicted structures. (**B**) Rank of proteins with nucleosome histone binding capabilities based on SF scores. The graphical line represents the normalized SF scores. The red lines indicate the proteins with known PDB structure interacting with nucleosome acidic patch. Highlighted on top are RING-family ubiquitin E3 ligases with reported nucleosome complex structure. (**C**) Violin plots showing the SF scores across the four core histones H2A, H2B, H3, and H4. These plots depict the distribution of binding strength and binders across different histones.

In accordance with the file upload requirements of the AlphaFold server, we generated over 300 JSON files, each containing 20 folding pairs (Fig. 1A). Each folding pair consists of one full-length (less than 4053) nuclear protein and five elements from two molecules: H2A [human H2A type 2-C residues 12–119], H2B [human H2B type 1-K residues 32–125], H3 [human H3.2 residues 39–134], H4 [human H4 residues 21–103], and 601 Widom DNA [153-MER] reported in a human 153-bp DNA nucleosome (PDB: 6Y5E) [35]. For sequence lengths of nuclear protein from 4053 to 8000, we divided it into two parts based on the principle of minimizing disruption to the domain structure and then folded them with 153 bp nucleosomes, respectively. The model seed is set to 985866441 and ensured consistency across all jobs.

### Nucleosome binding score calculation

To effectively quantify and analyze large-scale predicted structure data, our approach focuses on molecular interactions, specifically amino acid contacting pairs, as illustrated in Fig. 1A. Initially, all structures showing pronounced steric clashes—flagged by the AlphaFold built-in “has_clash” metric—and any models with evident histone-related clashes were excluded from further analysis. We then collect pairs of atoms (one from the candidate protein and one from histone chains) that are within 5 Å for subsequent analysis. The formula employs two indices: *p*_*a*,*k*_ and *p*_*l*,*k*_, derived from AlphaFold’s predicted PAE and pLDDT scores, which reflect confidence in the relative positioning and local structure of residues, respectively. For *p*_*l*,*k*_, we take the raw pLDDT score as is, and for *p*_*a*,*k*_, we subtract rawPAE from 31.75 (typically, 31.75 represents the maximum raw PAE value obtained through prediction; since a higher raw PAE indicates a poorer outcome, this transformation aligns its trend with pLDDT, where a higher raw pLDDT denotes a better result). The product of these two indices is averaged before being assigned as the weight for the *k*th contacting pairs for each nuclear protein, and their sum constitutes the unnormalized strength factor (SF). The scaling factor, B, is the unnormalized SF for BARD1 (e.g. BARD1-nucleosome complex in PDB: 7E8I) [36] calculated in the way above. For each structure, the total weight is divided by B to standardize scores for comparison, resulting in the SF.

### PDB benchmarking data retrieval

We performed a search in the structure database on the NCBI website using the keyword, ((((“histone”[Protein Name]) AND 9:100[BioUnit Protein Molecule Count]) AND 2:2[BioUnit DNA Molecule Count])) AND “Homo sapiens”[Organism], successfully retrieving 322 nucleosome complex structures as of 7 August 2024. These structures were then batch-downloaded. After that, we employed Python scripts for detailed analysis, discarding non-human-derived binders. This process led to the identification of 75 proteins with distinct histone contacts within the nucleosome. Of these, 30 proteins were found to interact specifically with the nucleosome’s acidic patch region.

### Acidic patch analysis

**Figure 2.**
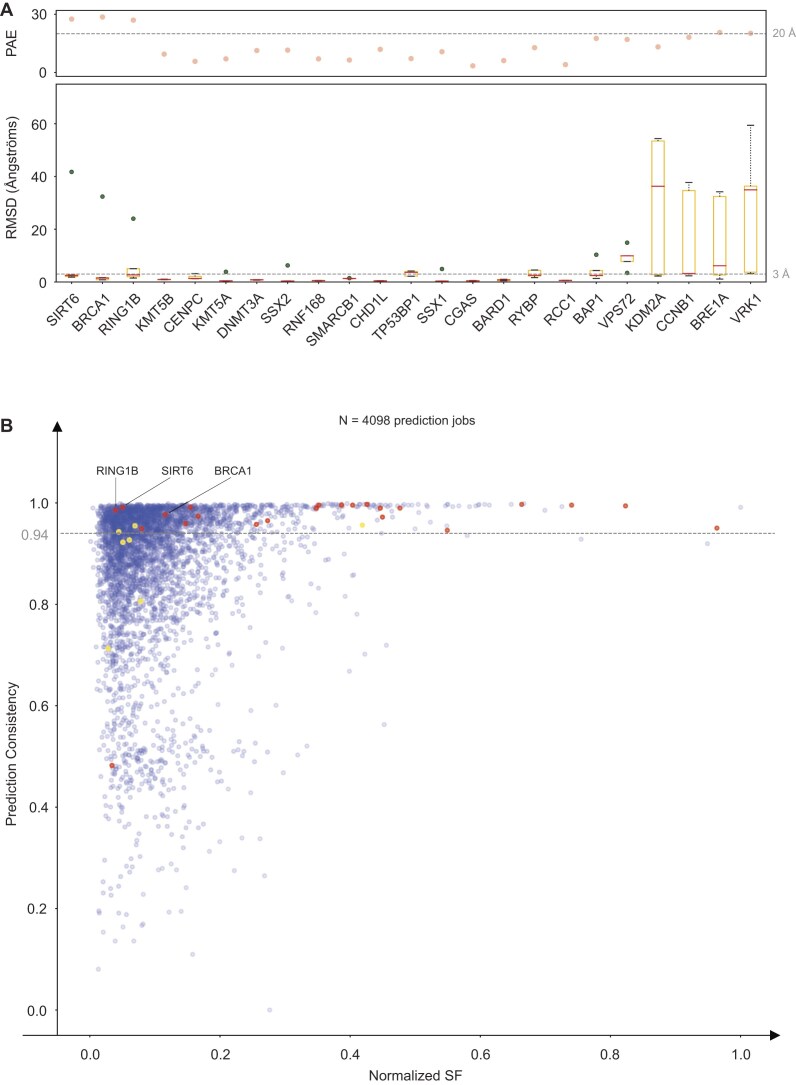
Prediction consistency and structural alignment of nucleosome acidic patch binders (**A**) RMSD of five model structures for each predicted nucleosome acidic patch binder, compared to their established structures at the binding region. Each dot represents the RMSD value for one model, with predicted aligned error (PAE) values displayed at the top of the chart. (**B**) Scatter plot analyzing prediction consistency against normalized SF for potential nucleosome binders. Red dots represent the 23 proteins accurately predicted, while yellow dots denote 7 inaccurately predicted proteins. A consistency threshold line at 0.94 delineates high-confidence predictions, with specific markers for SIRT6, RING1B, and BRCA1 highlighting their positions within the analysis.

The acidic patch region here is defined by E57, E62, E65, D91, E92, and E93 of human H2A type 2-C (Q16777). The classical acidic patch pocket (AP1) here is defined by E62, D91, and E93 and AP2 is defined by E62, D91, and E65 (Supplementary Fig. S2B). When performing sequence motif analysis, we first extracted amino acids on the binder chains having contacts with the acidic patch. Then, alignment was performed by extending 10 amino acids to the left and right, centering on the classic pocket region’s arginine or lysine. When performing benchmarking analysis, if interaction pairs at the acidic patch between AF3 predicted structures and experimentally solved structures are aligned well, we consider this candidate as positive hit. When performing prediction consistency analysis, the amino acids involving interactions on binders were first extracted by constraining within five angstroms of the centroid of acidic patch (AP1 and AP2). Then we calculated the pairwise root mean square deviation (RMSD) among them across five models, using mode value of RMSD as the estimate for each protein’s prediction consistency at acidic patch. The *y*-value of each point is defined as one minus the ratio of the absolute mode RMSD of each binder to the maximum RMSD in all binders (Fig. 2B). When a potential binder has multiple binding sequences at acidic patch, we selected the longest one; if lengths are identical, a sequence was chosen at random as the representative.

### Gene Ontology enrichment analysis

Gene Ontology (GO) enrichment analysis was conducted using the PANTHER Overrepresentation Test (released on 7 August 2024) accessible through the Gene Ontology website. The analysis employed the GO Ontology database version DOI: 10.5281/zenodo.12173881, released on 17 June 2024. Our gene IDs are provided in UniProt ID form, and the reference list included all human genes available in the database. Statistical significance of GO term enrichment was determined using Fisher’s exact test. To correct for multiple testing and control the false discovery rate (FDR), we applied the FDR calculation method provided by the PANTHER tool.

### Binder-nucleosome interaction region analysis

We roughly defined different combinations of contact regions between candidate binders and nucleosomes organized by histone type, with a total of 15 theoretical combinations. Based on the SF formula, we analyzed the interactions between candidate binders and four types of histones in all predicted data, extracted amino acid pairs having valid contacts, and counted the number of hits in each histone combination category.

### Calculation of ΔΔG

PyRosetta4 was used to calculate the ΔΔG (delta-delta-G), an estimate of the binding energy of a complex [37]. We first relaxed the AF3-predicted structural models in Rosetta by repacking the side chains and minimizing the energy within Cartesian coordinate space, applying coordinate constraints to prevent significant deviations from the input structures during these steps. Subsequently, the ΔΔG was calculated using the Rosetta relaxed structures. Among the five AF3 models generated, the one with the lowest Rosetta ΔΔG was selected as the representative for subsequent comparisons.

### Protein expression and purification

*Homo sapiens* ARID4A (1–150) or ARID4B (1–150) gene was synthesized by Synbiob (Tianjin) and cloned into pET28a-SUMO vectors and was expressed in *Escherichia coli*. The construct is sequentially linked from N-terminal to C-terminal by a 6 × His tag, a thrombin site, a small ubiquitin-like modifier (SUMO) tag, and ARID4A (1–150) or ARID4B (1–150). *Escherichia coli* cultures were grown at 37°C in the Minquan MQD-S1R shaking incubator at 220 rpm. When the optical density at 600 nm (OD_600_) reached 0.6–0.8, protein expression was induced by adding 0.5 mM IPTG at 37°C. Following induction, the cultures were incubated for an additional 5 h. Cells were then harvested by centrifugation (3030 g, 4°C, 25 min), and the supernatant was discarded. Cell pellets were rapidly frozen and immediately stored at −80°C.

*Homo sapiens* ARID4A (1–150) and ARID4B (1–150) were purified using the same method. *Escherichia coli* cells expressing ARID4A (1–150) or ARID4B (1–150) were resuspended in pre-cooled lysis buffer (20 mM Tris–HCl, pH 8.0, 500 mM NaCl, 20 mM imidazole) and all operations were at ice or 4°C. The resuspended bacterial cells were lysed using a high-pressure homogenizer at 800–900 bar. The lysate was then subjected to high-speed centrifugation at 30 966 g for 1 h at 4°C. The supernatant was collected and incubated with pre-equilibrated self-packed nickel beads at 4°C for 1 h. The beads were washed with 6 column volumes (CV) of lysis buffer, and the target protein was eluted with 2 CV of elution buffer (20 mM Tris–HCl, pH 8.0, 500 mM NaCl, 250 mM imidazole). Protein purity was assessed by sodium dodecyl sulfate–polyacrylamide gel electrophoresis (SDS–PAGE). While stirring, the collected eluate was diluted with dilution buffer (20 mM Tris–HCl, pH 8.0) to adjust the NaCl concentration to 50 mM. The diluted solution was filtered through a 0.2 μm PVDF membrane and concentrated using Amicon 10 000 MWCO centrifugal filter unit (Millipore). The concentrated samples were aliquoted, flash-frozen in liquid nitrogen, and immediately stored at −80°C for future use. The plasmid containing ARID4A (1–150) or ARID4B (1–150) mutant gene was generated by site-directed mutagenesis and purified as described earlier.

*Homo sapiens* SET8/KMT5A (194–393) was expressed and purified as previously reported [11]. The expression and purification of *Xenopus laevis* histones, the single-chain form of human H2A–H2B dimer (scH2A–H2B) fused with a protein A, and 601 Widom DNA (147-MER) were performed as previously described [36, 38–40]. The scH2A–H2B acidic patch consists of H2A residues E57/E62/E65/D73/D91/E92/E93A and H2B residues E106/E114A. The DNA for nucleosome pulldown assay is biotinylated 162 base-pair DNA, a 15N0 modified version of 601 Widom DNA, purified through polymerase chain reaction (PCR) followed by anion exchange chromatography using a loading buffer of 20 mM Tris–HCl (pH 8.0) and 500 mM NaCl, and an elution buffer of 20 mM Tris–HCl (pH 8.0) and 1.5 M NaCl.

*Homo sapiens* RNF168 and UbcH5c gene were obtained from a complementary DNA library and subsequently cloned into the pET28a vectors. Various truncations of the RNF168 were generated by PCR mutagenesis and cloned into the pET28a vector. The expression vector for the RNF168 and UbcH5c fusion gene was constructed using homologous recombination. The constructs include an N-terminal 6 × His tag followed by a thrombin cleavage site. *Escherichia coli* cultures were grown at 37°C in the Minquan MQD-S1R shaking incubator at 220 rpm. When the temperature was lowered to 16°C and the optical density at 600 nm (OD_600_) reached 0.6–0.8, protein expression was induced by adding 0.5 mM IPTG. After 15–18 h of induction, cells were harvested using the method above, flash-frozen, and stored for subsequent purification.

*Homo sapiens* RNF168 and UbcH5c were purified in a similar manner. The truncated form of RNF168 was first purified using nickel-affinity chromatography with a lysis buffer containing 25 mM Tris–HCl (pH 8.0), 500 mM NaCl, 10 μM ZnCl_2_, 25 mM imidazole, and 5 mM β-mercaptoethanol (β-ME), and an elution buffer with 25 mM Tris–HCl (pH 8.0), 300 mM NaCl, 10 μM ZnCl_2_, 250 mM imidazole, and 5 mM β-ME. This was followed by a single step of size-exclusion chromatography using a buffer composed of 25 mM Tris–HCl (pH 8.0), 300 mM NaCl, and 10 μM ZnCl_2_, with eluates collected in fractions. Fractions containing high-purity protein, as confirmed by SDS–PAGE, were pooled, flash-frozen in liquid nitrogen, and stored at −80°C for future use. The UbcH5c was initially purified using nickel-affinity chromatography with a lysis buffer of 25 mM Tris–HCl (pH 8.0), 500 mM NaCl, 25 mM imidazole, and 5 mM β-ME, and an elution buffer of 25 mM Tris–HCl (pH 8.0), 500 mM NaCl, 250 mM imidazole, and 5 mM β-ME. This was followed by cation exchange chromatography, using a loading buffer of 25 mM Tris–HCl (pH 8.0) and 150 mM NaCl, and an elution buffer of 25 mM Tris–HCl (pH 8.0) and 2 M NaCl. The RNF168-UbcH5c fusion protein was first purified using nickel-affinity chromatography with a lysis buffer of 25 mM MES–NaOH (pH 6.0), 500 mM NaCl, 10 μM ZnCl_2_, 25 mM imidazole, and 5 mM β-mercaptoethanol (β-ME), and an elution buffer of 25 mM MES–NaOH (pH 6.0), 300 mM NaCl, 10 μM ZnCl_2_, 250 mM imidazole, and 5 mM β-ME. This was followed by cation exchange chromatography using a loading buffer of 25 mM MES–NaOH (pH 6.0) and 200 mM NaCl, and an elution buffer of 25 mM MES–NaOH (pH 6.0) and 2 M NaCl. Finally, the sample was subjected to size-exclusion chromatography with a buffer of 25 mM MES–NaOH (pH 6.0), 300 mM NaCl, and 10 μM ZnCl_2_. The RNF168'-RNF168-UbcH5c fusion protein was purified first by nickel-affinity chromatography using a lysis buffer of 25 mM MES–NaOH (pH 6.0), 1 M NaCl, 10 μM ZnCl_2_, 25 mM imidazole, and 5 mM β-mercaptoethanol (β-ME), and an elution buffer of 25 mM MES–NaOH (pH 6.0), 1 M NaCl, 10 μM ZnCl_2_, 250 mM imidazole, and 5 mM β-ME. This was followed by cation exchange chromatography with a loading buffer of 25 mM MES–NaOH (pH 6.0), 300 mM NaCl, and an elution buffer of 25 mM MES–NaOH (pH 6.0), 2 M NaCl. The final purification step was size-exclusion chromatography with 25 mM MES–NaOH (pH 6.0), 1 M NaCl, and 10 μM ZnCl_2_ as the buffer. Purity was assessed as described previously, with high-purity fractions pooled, flash-frozen in liquid nitrogen, and stored at −80°C for future use.

### Assembly of nucleosome core particle

Histones and DNA were expressed and purified as described earlier. Nucleosome core particles were assembled using the classical salt gradient dialysis method [38]. The assembled nucleosomes were concentrated and stored at 4°C. Quantification of nucleosomes was performed by measuring the DNA content of the samples.

### Electrophoretic mobility shift assay

Purified wild-type 147 bp nucleosome core particles at a concentration of 100 nM were incubated with increasing concentrations of the binder at 4°C for 15 min in 8 μL of reaction buffer, containing 20 mM Tris–HCl (pH 8.0) and 50 mM NaCl. The concentration of nucleosome core particle and reaction volume of RNF168 were 50 nM and 20 μL, respectively. The reaction mixtures were then loaded onto a 6% native PAGE gel for electrophoresis. After electrophoresis, the gels were stained with ethidium bromide (EtBr) and imaged using a Tanon 1600 gel imaging system.

### Histone dimer and nucleosome pulldown assay

For histone dimer pulldown assay, the single chain H2A–H2B dimer with a protein A tag (wild-type or mutant) was immobilized on IgG beads to act as the bait, and ARID4A/4B 1–150 (wild-type or mutant) was introduced as the prey. Bait and prey were mixed at a 1.5: 1 molar ratio (prey : bait), and beads were added in slight molar excess over the bait; the mixture was incubated in binding buffer (20 mM Tris–HCl, 150 mM NaCl, pH 8.0) at 4°C for 1 h, after which the beads were washed four times with the same binding buffer. Elution was then carried out by incubating the beads with elution buffer (0.1M Glycine, pH 3.0) for 20 min, and the resulting eluate was examined by SDS–PAGE. The nucleosome pulldown assay represents a modified version of Horikoshi *et al.* [41], with both the binding buffer and wash buffer corresponding to 20 mM HEPES–NaOH (pH 7.5), 50 mM NaCl, 0.2 mM ethylenediaminetetraacetic acid, 5% glycerol, 0.1% NP-40, and 1 mM DTT.

### Cryo-EM sample preparation

The formation of the RNF168'-RNF168-UbcH5c (E3'-E3-E2 in single chain) in complex with nucleosome was achieved by mixing the components and dialyzing them into a buffer containing 10 mM HEPES–NaOH (pH 7.5), 10 μM ZnCl_2_, and 4% glycerol. The mixture was then centrifuged at 4°C and 5867 g for 10 min to remove any precipitates. A density gradient centrifugation buffer was prepared using a top buffer containing 10 mM HEPES–NaOH (pH 7.5), 10 μM ZnCl_2_, and 5% sucrose and a bottom buffer containing 10 mM HEPES–NaOH (pH 7.5), 10 μM ZnCl_2_, 20% sucrose, and 0.15% glutaraldehyde. The RNF168'-RNF168-UbcH5c complex with nucleosome was then mildly cross-linked within the prepared density gradient centrifugation buffer and centrifuged at 4°C, 222 200 g for 20 h. The distribution range of the sample was assessed by Native PAGE, and high-quality fractions were collected, concentrated, and exchanged into a sucrose- and glutaraldehyde-free buffer (10 mM HEPES–NaOH, pH 7.5). Samples at varying concentrations were prepared for subsequent cryo-EM analysis.

The RNF168'-RNF168-UbcH5c complex with nucleosome was vitrified on Quantifoil R1.2/1.3 300-mesh gold (Au) grids, which were glow-discharged for 60 s in an H_2_/O_2_ gas mixture. A 3 μL aliquot of the sample was applied to each side of the grid, allowed to incubate for 10 s, then blotted with Vitrobot filter paper for 3.5 s at blot force 3. The grids were immediately plunge-frozen in liquid ethane using a Vitrobot Mark IV (FEI Company) set to 4°C and 100% humidity.

### Cryo-EM data collection and processing

Cryo-EM data were collected at the Center for Biological Imaging, Institute of Biophysics, Chinese Academy of Sciences (CBI, IBP, CAS), using a Titan Krios 300 kV transmission electron microscope equipped with a K3 direct electron detector (no energy filter). The total electron dose was set to 50 e^−^/Å², with a pixel size of 1.07 Å. Defocus values were preset between −1.8 and −2.2 μm. Each image was captured over 32 frames, with motion correction applied in real-time during acquisition. A total of 4652 movies were collected (Supplementary Fig. S5A).

The collected set of 4652 cryo-EM micrographs was initially processed in Relion 3.0 [42]. Automated particle picking yielded 5 941 000 particles, which were extracted with binning (bin4) to save storage space. After 2D classification, 4 760 742 particles remained (Supplementary Fig. S5B). Two rounds of 3D classification were performed, discarding unstable nucleosomes and free nucleosomes without protein binding. A mask was then applied to screen for additional densities on the nucleosome surface, resulting in the removal of 15.2% of particles lacking this density. The remaining 1 557 912 particles constituted the total nucleosome complex dataset for further analysis. In Relion 3.0, nucleosome surface protein density was further masked and classified. During mask creation, parts of the histone core were retained as a reference to compare histone and surface protein densities in the classification results. Through multiple rounds of refinement, homogeneous particles were retained, ultimately yielding 50 851 particles for 3D refinement, producing a 4.6 Å electron density map (Supplementary Fig. S5C, Map1). In CryoSPARC 4.4.1 [43], a focused 3D classification of the nucleosome complex retained only the most homogeneous class. This classification resulted in slightly reduced surface density compared to Relion 3.0, but with improved resolution. Further subclassification did not yield significant improvements, and the final set of 58 069 particles produced a 3.9 Å electron density map, which was subsequently post-processed using EMready 2.0 [44]. (Supplementary Fig. S5C, Map2).

### Model building and refinement

The refined map was used for model building. The crystal structures of the nucleosome (PDB ID: 3LZ0) [45] and RNF168 (PDB ID: 4GB0) [46] were fit into the map to create the initial model using ChimeraX 1.7.1 [47]. This preliminary model was then manually adjusted in Coot 0.8.9 [48], followed by automated refinement and validation in Phenix 1.21.1 [49]. The refinement and validation statistics of models are shown in Supplementary Table S3.

### Analytical ultracentrifugation

The samples for analytical ultracentrifugation were prepared by concentrating or diluting to achieve an OD of 0.8, in a buffer containing Tris–HCl (pH 7.5) and 300 mM NaCl. Sedimentation experiments were conducted on a Beckman Coulter ProteomeLab XL-I analytical ultracentrifuge with an An-60Ti rotor (Beckman). Absorbance at 280 nm was recorded continuously in scan mode as the sample sedimented within aluminum dual-sector cells at 16°C, 58 000 rpm, for 8 h to reach sedimentation equilibrium.

### *In vitro* ubiquitination assay

Sequentially add each reagent and protein to the reaction system in the following order: 10 mM MgCl_2_, 1 μM ZnCl_2_, 1 mM TCEP, 3.2 μM Uba1, 8 μM UbcH5c, 8 μM RNF168, 20 μM H2A–H2B dimer (or 20 μM nucleosome), 100 μM ubiquitin, and 3 mM ATP in the reaction buffer. The reaction buffer, consisting of 50 mM Tris–HCl (pH 7.5) and 100 mM NaCl, is used to adjust the total reaction volume to 50 μL. ATP should be added last. Before adding ATP, take a 5 μL sample of the pre-reaction mixture as the 0-time point. After ATP addition, initiate the reaction by incubating the mixture in a 32°C water bath. Take 5 μL samples at 15, 30, and 60 min, immediately quenching each sample by adding 5 μL of 8 M urea and SDS loading buffer. Denature the samples by heating at 100°C for 5 min in a metal bath to terminate the reaction. Analyze the samples using SDS–PAGE.

### Visualization creation, programming, and manuscript refinement

Figures and tables were crafted using Adobe Illustrator, PowerPoint, Python, and UCSF ChimeraX. ChatGPT was utilized for code development and manuscript enhancement.

## Results

### Prediction of nucleosome binding proteins using AlphaFold3 web server

AlphaFold3 (AF3) has recently been made available as a web server [31], although it has not yet been widely tested for its ability to predict nucleosome binding proteins. To explore its potential in this area, we analyzed 7655 human nuclear proteins for their binding affinity to a single nucleosome core particle using the AF3 webserver, generating 38 390 structural models (Fig. 1A). A computational approach was employed to quantify protein-nucleosome interactions, specifically targeting amino acid pair interactions. The SF value, a composite metric to evaluate these interactions, integrates the AlphaFold confidence parameters, PAE and predicted local distance difference test (pLDDT), with the 5 Å distance constraints (see the “Materials and methods” section and Fig. 1A). For each of the 7653 proteins, SF scores were obtained from five structural models per prediction. The highest SF score from these models was used to rank the proteins, creating a prioritized list of potential nucleosome-binding proteins based on their calculated SF values (Fig. 1B and Supplementary data). To assess the biological relevance of these predictions, we performed GO enrichment analyses on the top 149 hits identified using the Kneedle algorithm, revealing that the most enriched terms were associated with chromatin functions, supporting the alignment of predicted nucleosome binders with relevant biological roles (Supplementary Fig. S1A). The predicted structural database is available on web server (http://bigdata.ibp.ac.cn/ncpbindersdatabase-app) with an interactive interface (Supplementary Fig. S1B).

### Assessment of AlphaFold-guided prediction dataset

To evaluate the accuracy of our predictive approach, we compared our predicted structures with known nucleosome binding proteins that have documented structures in the Protein Data Bank (PDB) (Supplementary Table S1). Notably, among 75 histone-binding proteins, 20 interacting with partially unfolded nucleosomes or histone tails are excluded from our prediction dataset because our algorithm uses fully wrapped, tailless nucleosomes as the binding template (Supplementary Fig. S1C and D, and Supplementary Table S1). This method successfully predicted 56% (31/55) of proteins with established histone binding capabilities and 77% (23/30) of proteins known to bind the nucleosome acidic patch. We also explored the binding hotspots and distribution of binding patterns among the four core histones. Histones H2A and H2B were most frequently targeted (Fig. 1C), aligning with the observation that the acidic patch is the primary binding interface [18, 50]. Conversely, H3 and H4 were less frequently involved in nucleosome binding but exhibited stronger interactions, as indicated by their higher SF values (Fig. 1C and Supplementary Fig. S2A). Analyses of the usage of residues in the H2A–H2B acidic patch and the H2B C-helix during binding revealed that arginine and lysine were predominantly involved in acidic patch binding (Supplementary Fig. S2B and C), while acidic residues (Glu, Asp) and hydrophobic residues (Leu, Ile, Phe) played more significant roles in the H2B C-helix binding (Supplementary Fig. S2D). These results suggest the consistency of AF3-based predictions with the established physical and chemical principles governing nucleosome-protein interactions, such as salt bridge recognition.

Among the 23 proteins accurately predicted to interact with the nucleosome acidic patch, 20 exhibit relatively high SF scores, with their best models aligning with reported structures (Fig. 2A). Interestingly, SIRT6, the RING-type ubiquitin ligases RING1B and BRCA1 exhibited low SF scores, likely due to exceptionally high PAE values (Fig. 2A). Despite the low SF scores, predicted models for these proteins display high repeatability and alignment with known structures, indicating that low SF scores might imply the binding of dynamic rather than lack of interactions (Fig. 2A). To assess this further, we measured the RMSD across five model structures for 4086 proteins, evaluating the prediction consistency and binding potential to the nucleosome acidic patch (Fig. 2B). Remarkably, 22 out of 23 proteins achieved high prediction consistency scores, ranking them in the top 6% of all analyzed proteins (Fig. 2B). These results emphasize that high prediction consistency can effectively prevent misinterpretations of low SF scores, which might otherwise suggest a lack of interaction.

### Discovery of ARID4A and ARID4B as novel nucleosome-binding proteins

**Figure 3.**
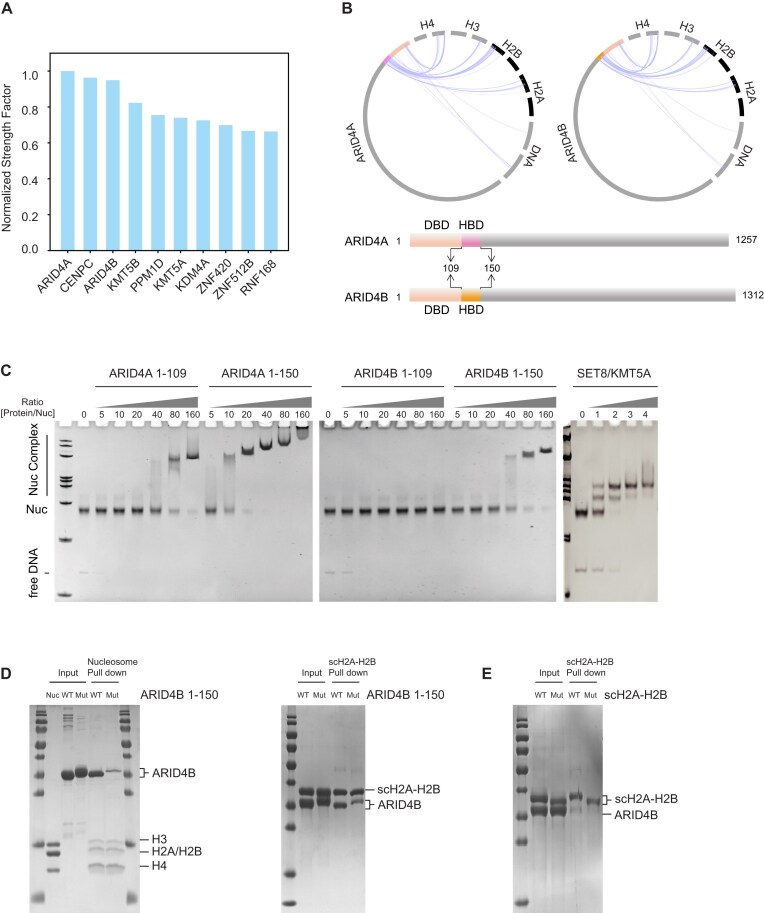
Novel insights into ARID4A and ARID4B as nucleosome-binding proteins. (**A**) Ranking of the top 10 hits based on the SF scores with nucleosome binding capabilities. (**B**) Diagrams illustrate specific interaction pairs between the nucleosome and ARID4A or ARID4B, highlighting DBD and HBD in each protein (top). Schematic representations of ARID4A and ARID4B display interaction domains and nucleosome binding regions (bottom). (**C**) EMSA validating the nucleosome binding capabilities of ARID4A, ARID4B, and SET8/KMT5A in a stoichiometry-dependent manner. The gel images demonstrate the interaction strength and specificity of each protein with the nucleosome. (**D**, **E**) Pull-down analyzing the nucleosome acidic patch binding capabilities of ARID4A/4B HBD. The single-chain H2A–H2B dimer (scH2A–H2B) and nucleosome were immobilized to pull down the wild-type or mutant ARID4B 1–150. scH2A–H2B mutations: H2A E57A/E62A/E65A/D73A/D91A/E92A/E93A, H2B E106A/E114A. ARID4B 1–150 mutations: K138A/K139A/R142A/R144A/R144A.

Among the top 10 ranking proteins, four (CENPC, KMT5B, KMT5A, and RNF168) are established nucleosome-binding proteins with well-documented structures [11, 12, 51–53]. In contrast, ARID4A and ARID4B, known for their AT-rich interactive domains, were not previously identified as nucleosome-binding proteins (Fig. 3A). The homologous proteins ARID4A and ARID4B are critical transcription factors that regulate the cell cycle, embryonic stem cell differentiation, and mediating epigenetic modifications [54, 55]. ARID4A/4B are potential nucleosome binders given that their Tudor, ARID, and chromobarrel domains exhibit binding activity to DNA or histone modification [56–58]. Predictive binding analyses indicate that both ARID4A and ARID4B engage nucleosomes using their N-terminal 1–150 residues, a result validated by EMSA (Fig. 3B and C). As a positive control, KMT5A (residues 194–393) exhibited similar EMSA results, validating our predictive approach (Fig. 3C and Supplementary Fig. S2E). ARID4A/4B achieve high prediction consistency scores, suggesting that the predicted structures are consistent across different models (Supplementary Fig. S3A and B). Detailed analysis mapped nucleosomal DNA interactions to the ARID4A/4B DNA-binding domain (DBD; residues 1–109) [56, 57], while histone binding localized to the histone-binding domain (HBD; residues 110–150) (Fig. 3B and Supplementary Fig. S3C and D). Deletion of the HBD (Δ110–150) severely impaired nucleosome binding, confirming its essential role in ARID4A/4B-nucleosome interactions (Fig. 3C). To pinpoint key interactions involved by HBD, we mutated HBD residues predicted to engage the nucleosome acidic patch (Supplementary Fig. S3C and Fig. 3D). Pull-down assays showed significantly reduced binding of this ARID4B HBD mutant to both nucleosomes and H2A–H2B dimers (Fig. 3D). This phenotype mirrored binding defects observed with the H2A–H2B acidic patch mutant (Fig. 3E) and ARID4A mutant (Supplementary Fig. S3E), indicating that these residues mediate ARID4A/4B-acidic patch interactions. This discovery highlights the ability of our ranking system to identify novel nucleosome binders and resolve interaction mechanisms.

### Nucleosome binding analyses of RING-type ubiquitin E3 ligases

**Figure 4.**
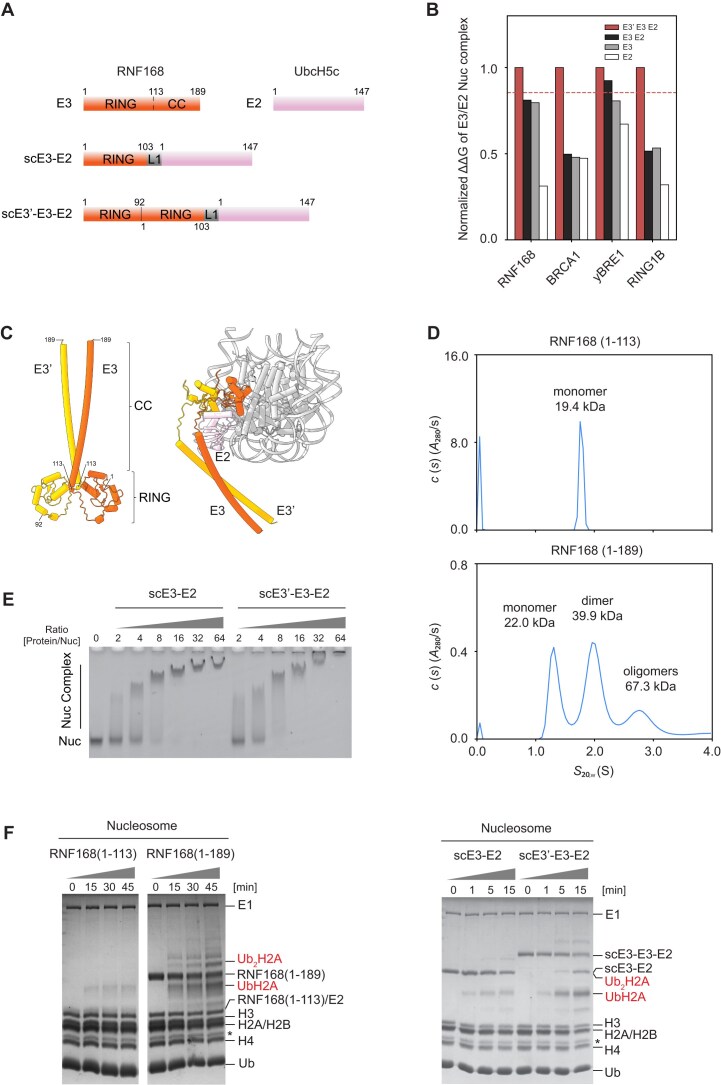
Comprehensive analysis of ubiquitin E3 ligase RNF168. (**A**) Schematic representation of RNF168 and UbcH5c constructs. Displays of scE3-E2 and scE3'-E3-E2 where the RING domain of RNF168 is connected to UbcH5c or itself, illustrating different engineering strategies. L1 loop consists of residues GSGSR. (**B**) Rosetta ΔΔG analysis of RING-domain ubiquitin E3 ligase-containing complexes predicted by AlphaFold3. The comparative stability of different ubiquitin E3 ligase configurations in complex of nucleosomes is normalized against the ΔΔG value of E3'-E2-E2 complex. The red dotted line marks the normalized ΔΔG value of the RNF168 scE3'-E3-E2 construct utilized in the experiments. (**C**) AlphaFold models suggesting the predicted dimerization of RNF168 in nucleosome-free and nucleosome-bound states, highlighting the flexibility and structural adaptability of the RNF168 molecule. AlphaFold-Multimer and AlphaFold3 are used to predict structure of E3'-E3 dimer or nucleosome-bound E3'-E3-E2, respectively. (**D**) Analytical Ultracentrifugation (AUC) data illustrating the oligomeric states of RNF168, distinguishing between monomer, dimer, and higher-order oligomers. (**E**) EMSA of nucleosome binding by scE3-E2 and scE3'-E3-E2. (**F**) *In vitro* ubiquitination activity assessment. Coomassie-stained gel showing ubiquitination activity of RNF168, scE3-E2, and scE3'-E3-E2 complexes. Asterisk (*) denotes contaminant from histone degradation.

The observed low SF scores for RING-family ubiquitin E3 ligases, such as RING1B, BRCA1, and BRE1A, suggest potential structural flexibility or local dynamics within these complexes (Fig. 1B). This aligns with previous studies that E3 ligases need to form complexes with other binding partners, including dimer subunits of E3 ligases and RING-family ubiquitin E2 ligases, to enhance nucleosome interactions [26–28, 59]. AlphaFold3 effectively predicted structures of E3-E2-nucleosome complexes, including those of RING1B, BRCA1, RNF168, and yeast BRE1 (termed yBRE1), yet it failed to predict the structure of the human BRE1A-BRE1B-RAD6A complex. To eliminate the impact of extreme PAE values, we employed Rosetta ΔΔG calculation to analyze the energetic stability of different complexes, such as yBRE1 (E3), RAD6 (E2), yBRE1-RAD6 (E3-E2), and yBRE1-yBRE1-RAD6 (E3'-E3-E2) (Fig. 4A and B, and Supplementary Table S2). Our data suggested that complex formation significantly improved nucleosome interactions, as seen with yBRE1-RAD6 and yBRE1-yBRE1-RAD6 (Fig. 4B). A similar enhancement in binding was observed for E3 ligases RING1B and BRCA1 when partnered, corroborating with established structures (Fig. 4B).

### Role of RNF168 dimerization in nucleosome interaction

RNF168, a well-known E3 ubiquitin ligase, is recognized for its role in ubiquitinating H2AK13/K15 through a complex with UbcH5c [53, 60]. To stabilize the RNF168-nucleosome complex for structural analysis, specific methods like fusing E3/E2 to the H2A–H2B dimer or crosslinking E3/E2 to H2A at K13/K15 are utilized [53, 61]. While it is unclear if RNF168 dimerizes within the nucleosome context, AlphaFold predictions suggest that RNF168 (1–189) can dimerize both with and without nucleosomes (Fig. 4C). AUC confirms that RNF168 (1–189) forms dimers, dependent on both the RING domain and the coiled-coil region, aligning with previous findings [59] (Fig. 4D). Enhanced ΔΔG scores from RNF168'-RNF168-UbcH5c (E3'-E3-E2) and RNF168-UbcH5c (E3-E2) complexes indicate significantly improved nucleosome interactions, supported by EMSA results (Fig. 4E). Notably, direct fusion of two RNF168 (1–189) monomers is sterically prohibited due to the long distance between their termini in the dimeric model (Fig. 4C). To resolve this spatial constraint, we engineered a single-chain construct scE3'-E3-E2 by fusing RNF168 (1–92) to the scE3-E2 scaffold via a flexible five-residue linker. This design maintains the functional dimer geometry (Fig. 4A and C). Predictive modeling indicated that the scE3'-E3-E2 construct shares a similar binding mode with the scE3-E2 construct (Supplementary Fig. S4A). The observed increase in ΔΔG scores suggests enhanced nucleosome binding (Fig. 4B). Collectively, these results imply that RNF168 dimerization within a nucleosome context may facilitate robust RNF168-nucleosome interactions.

### Structure of RNF168 dimer bound to nucleosome

**Figure 5.**
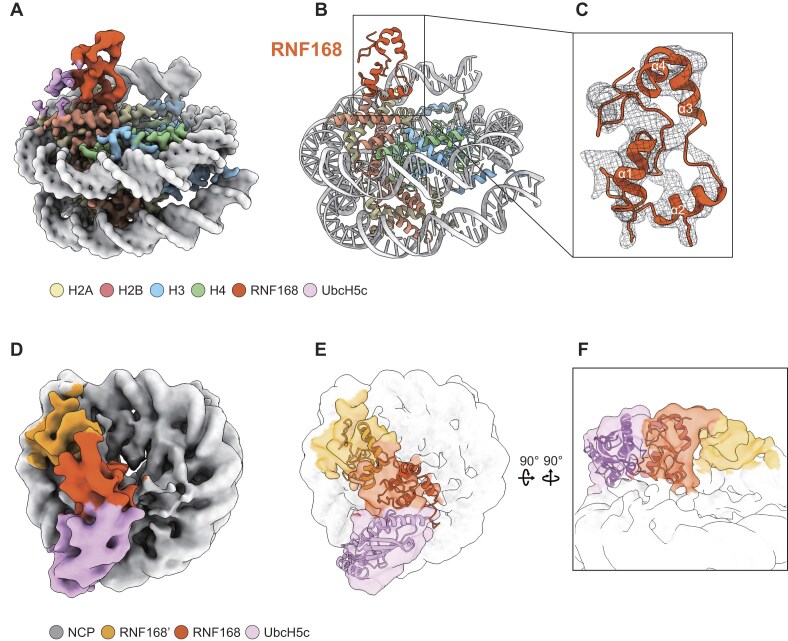
Cryo-EM structure of the dimerized RNF168 RING domain bound to nucleosome. (**A**) Cryo-EM density map of the scE3'-E3-E2 complex with nucleosome. Displayed density include subunit of RNF168 RING domains (E3) and UbcH5c (E2). (**B**) Cartoon representation of the scE3'-E3-E2–nucleosome complex. E3 RING domain (orange) is boxed and highlighted in panel (C). (**C**) cryo-EM density map of E3 RING domain fitted with solved RNF168 structure (PDB: 4GB0) [46]. (**D**) Cryo-EM density map of dimerized RNF168 RING domains and UbcH5c within the scE3'-E3-E2 complex. Cryo-EM density map of scE3'-E3-E2–nucleosome complex fitted with to the AF3-predicted scE3'-E3-E2-nucleosome structure (**E**) or RNF168-UbcH5c-nucleosome complex cryo-EM structure (PDB: 8SN1) (**F**) [53].

To assess whether the scE3'-E3-E2 construct might facilitate the complex structure study, we next compare this newly designed construct to scE3-E2 used in prior structural studies [53, 61]. EMSA confirmed that both constructs exhibit comparable nucleosome-binding efficiency (Fig. 4E). Despite that, scE3'-E3-E2 presents a higher ubiquitination activity than scE3-E2 (Fig. 4F). Consistent with this result, an increased activity is observed for dimeric RNF168 (1–189) over monomeric RNF168 (1–113), demonstrating that RNF168 dimerization enhances catalytic activity (Fig. 4F). Importantly, scE3'-E3-E2 exhibited enhanced stability compared to scE3-E2 during nucleosome complex preparation and cryo-EM data collection (Supplementary Fig. S4B and C), enabling structure determination at 3.9 Å resolution (Fig. 5A–C and Supplementary Figs S5 and S6). In our E3'-E3-E2-nucleosome cryo-EM structure, the E3 subunit aligns precisely with its counterpart in the RNF168-UbcH5c-nucleosome complex (PDB: 8SN1) [53], enabling unambiguous assignment of adjacent densities to E2 and E3' (Fig. 5C–F, Supplementary Fig. S6E). Structural analysis confirms that the E3–E2 nucleosome-binding mode closely matches prior observations (Supplementary Fig. S7) and aligns with AlphaFold3-predicted scE3'-E3-E2 architecture (Fig. 5D–F). Although limited local resolution at the E3'-E3 interface precludes detailed modeling, the map reveals E3' contacts with both the H2A C-terminal tail and SHL-6.5 DNA (Fig. 5D–F). These interactions suggest RING domain-mediated enhancement of nucleosome binding. Critically, our study presents the first cryo-EM structure of a RING E3-E2 complex bound to native nucleosomes lacking covalent E2 fusion, revealing how RNF168 dimerization facilitates substrate engagement.

## Discussion

Nucleosome-binding proteins are essential for regulating chromatin architecture and gene expression by modulating the interaction between DNA and histones. They play a pivotal role in fundamental cellular processes such as transcription, replication, and DNA repair by influencing nucleosome maintenance. In pursuit of discovering new nucleosome binders, we developed an *in silico* screening approach and assessed its prediction accuracy and consistency against known nucleosome acidic patch interactors as benchmarks. This method successfully identified ARID4A and ARID4B as novel nucleosome-binding proteins and demonstrated the dimerization of RNF168, a member of the RING-family ubiquitin E3 ligases, with structural validation provided by cryo-EM analysis. Notably, the engineered scE3'-E3-E2 construct may introduce non-physiological interactions due to forced E3'–E3 dimerization. Although we observe enhanced ubiquitination activity in dimeric constructs (Fig. 4F), we cannot exclude the possibility that endogenous RNF168 operates as a monomer under certain cellular conditions. Future studies using endogenous dimerization interfaces will clarify the physiological relevance of RNF168 dimerization.

The *in silico* screening procedure offers a rapid and effective means to discover and characterize nucleosome-binding proteins. It adapts well to the analysis of structural data across various interaction pairs involved in complexes. Using this strategy, we developed a scalable web tool that analyzes protein/DNA molecule structures, accommodating diverse file types like PDB, CIF, or JSON. This tool allows users to upload structures predicted by AlphaFold2, AlphaFold3, or experimentally resolved structures for in-depth analysis. Users can specify analytical parameters such as chain IDs, enabling control over the interaction regions to be analyzed. Moreover, our methodology extends to interaction interface analysis involving amino acids or nucleotides, providing flexibility in the parameters. For example, the acidic patch contacts, often overlooked in resolved structures, can be discerned with high confidence in predicted structures of proteins like SMARCA5 (PDB: 8V4Y) [62] or MEN1 (PDB: 8GPN) [63]. The tool also facilitates the analysis of nucleosomal DNA-binding proteins, including pioneer transcription factors and zinc finger proteins, broadening the scope of potential structural insights. By mining this data and employing similar approaches to screen for other related binding proteins, new protein–protein interaction networks could be uncovered.

Predicting dynamic structures remains a challenge for AlphaFold. In this study, AF3 occasionally misplaces sequences containing non-canonical arginine finger motifs targeting the nucleosome acidic patch. This limitation explains its unreliable prediction of certain nucleosome-binding proteins, whether they contain repetitive short peptide motifs, which compete for engagement with the acidic patch. This can be solved by expanding training datasets to include diverse interface geometries. Moreover, AF3-based computational framework introduced prediction bias. Addressing these constraints requires expanding training datasets to sample diverse interface geometries and integrating orthogonal experimental validation accounting for histone modifications and non-canonical nucleosome states. For example, study of Skrajna *et al.* [18] identified proteins exhibiting acidic patch sensitivity. Candidates with high SF scores in our ranking system provide orthogonal validation of nucleosome-binding capability.

Our data suggested that high prediction consistency can help correct misinterpretations of low SF scores, which may otherwise indicate a lack of interaction. The discrepancies are likely caused by structural flexibility or local dynamics within these proteins, highlighting the need for careful analysis of prediction consistency in nucleosome interaction analysis. Intriguingly, some proteins with lower prediction consistency still successfully bind nucleosomes, suggesting that factors beyond structural alignment contribute to nucleosome binding efficacy. Importantly, we identified proteins with low SF scores but medium-to-high prediction consistency, indicating their potential nucleosome interaction capabilities.

In the E3'-E3-E2-nucleosome complex structure, the primary E3 (E3) docks at the nucleosome acidic patch, whereas the secondary E3 (E3') interacts with the H2A C-terminal tail and SHL-6.5 DNA, a binding mode resembling BRE1–BRE1 (RNF20–RNF40) dimerization [26]. This dual interaction positions the E2 (UbcH5c) catalytic domain over H2A K13/K15, consistent with its known ubiquitination sites. The forced dimerization via the E3'-E3-E2 suggests a mechanism for site-specific ubiquitination at the H2A N-terminus. These data suggest that the RING-type ubiquitin ligases adopt two distinct E2 positioning modes for H2A, including the N-terminal targeting (as in RNF168–RNF168, BRE1–BRE1, and RNF20–RNF40) and C-terminal targeting (as in RING1B–BMI1 and BRCA1–BARD1) (Supplementary Fig. S7A). Positioning the E2 proximal to specific target lysine can be dictated by both homotypic and heterotypic E3 dimers, which engage the nucleosome in distinct binding modes.

Recent work suggests UDM1-mediated ubiquitin binding facilitates RNF168 recruitment [64]. Our data suggest a model where dimerization stabilizes nucleosome engagement while modulating ubiquitin chain elongation. Alternatively, dimerization might crosstalk with ubiquitin chain interaction, adding another layer of modulation to H2A K13/K15 modification. Although dimeric RNF168 constructs enhance nucleosome engagement and activity, the physiological relevance of endogenous dimerization requires further investigation. Limited local resolution at the E3'–E3 interface (Fig. 5D) precludes unambiguous determination of dimerization contacts. Future high-resolution studies or cellular assays will clarify this mechanism and resolve the interplay between dimerization and ubiquitin recognition.

## Supplementary Material

gkaf735_Supplemental_Files

## Data Availability

The predictome data for human nucleosome-binder complexes is available freely online at http://bigdata.ibp.ac.cn/ncpbindersdatabase-app. The generic web tool for structure analysis is accessible at http://bigdata.ibp.ac.cn/analysis-app. Atomic coordinates have been deposited in the Protein Data Bank under accession code 9KQ2, and the corresponding cryo-EM density maps are available in the Electron Microscopy Data Bank under accession code EMD-62494. The Python scripts used above are available freely on Zenodo (DOI: 10.5281/zenodo.14062135).

## Supplementary data

Supplementary data is available at NAR online.
