# Supplementary material for: AlphaFold-guided structural analyses of nucleosome binding proteins
Source: Nucleic Acids Res. 2025 Aug 6;53(14):gkaf735. doi: 10.1093/nar/gkaf735 (PMC12341937; doi:10.1093/nar/gkaf735)
Supplement: gkaf735_Supplemental_Files [file gkaf735_supplemental_files.zip › Supplementary_Figures_and_Tables_revised.pdf]

Figure S1

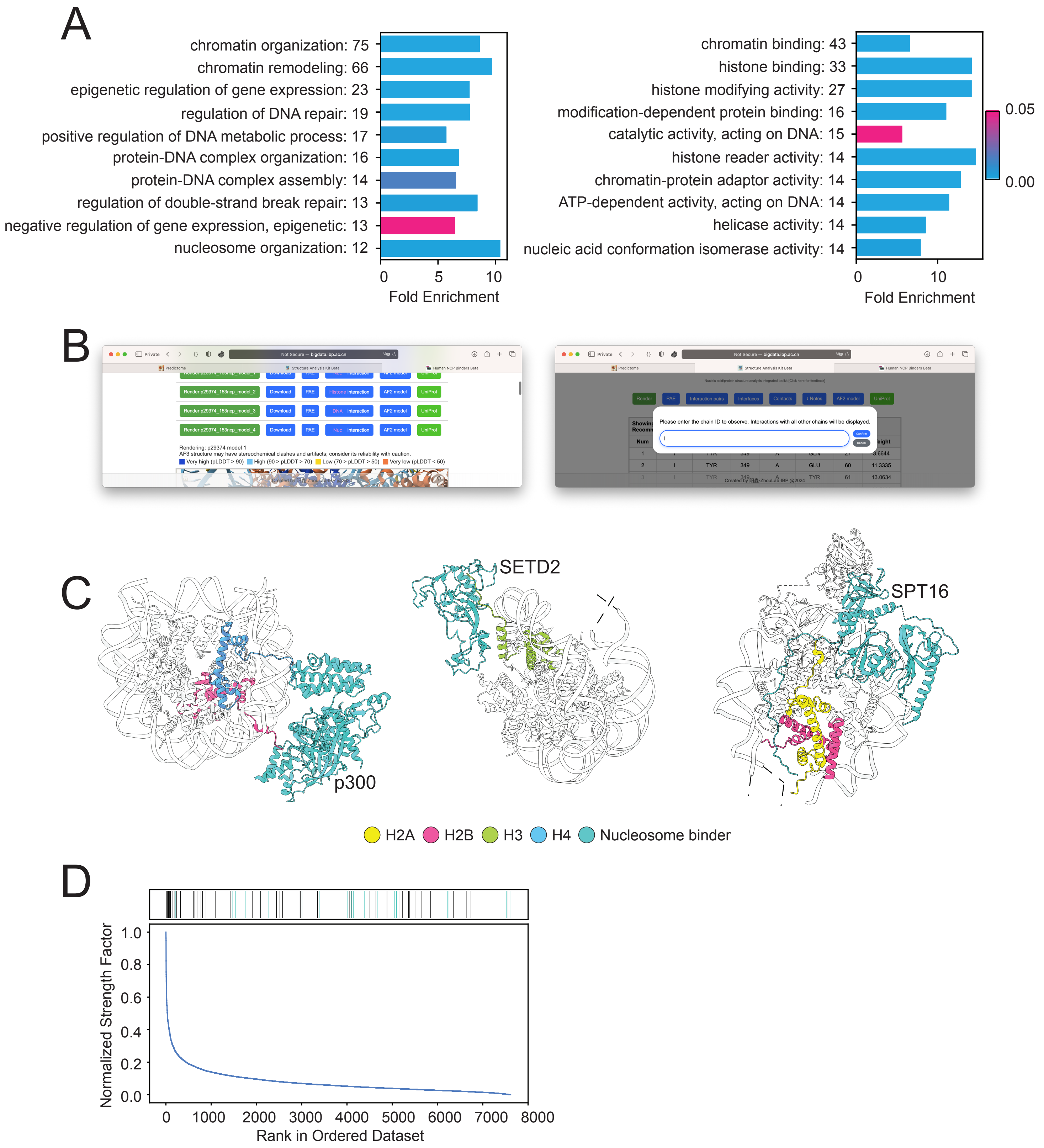
A
chromatin organization: 75
chromatin remodeling: 66
epigenetic regulation of gene expression: 23
regulation of DNA repair: 19
positive regulation of DNA metabolic process: 17
protein-DNA complex organization: 16
protein-DNA complex assembly: 14
regulation of double-strand break repair: 13
negative regulation of gene expression, epigenetic: 13
nucleosome organization: 12
0 5 10
Fold Enrichment
chromatin binding: 43
histone binding: 33
histone modifying activity: 27
modification-dependent protein binding: 16
catalytic activity, acting on DNA: 15
histone reader activity: 14
chromatin-protein adaptor activity: 14
ATP-dependent activity, acting on DNA: 14
helicase activity: 14
nucleic acid conformation isomerase activity: 14
0 10
Fold Enrichment
0.05
0.00
B
C
SETD2
SPT16
p300
H2A H2B H3 H4 Nucleosome binder
D
Normalized Strength Factor
1.0 0.8 0.6 0.4 0.2 0.0
0 1000 2000 3000 4000 5000 6000 7000 8000
Rank in Ordered Dataset

Figure S1. Online resources and analyses of predicted nucleosome binding proteins, related to Figure 1.

(A) GO enrichment analysis of biological process (left) and molecular function (right) applied to top 218 hits in screening. Number at the right of each category indicates hown many candidates in the entry. Bar length represents fold enrichment with a threshold set at 5; color intensity indicates False Discovery Rate (FDR) levels, highlighting high-confidence findings. The Kneedle algorithm was applied to the sorted data, resulting in 218 hits, which considered as candidates with strong nucleosome interaction.

(B) Screenshot of the online nucleosome binder prediction database (left) and analysis tool (right). It facilitates access to detailed predictions and interaction analyses, available online for broader scientific use.

(C) Illustrations of nucleosome-binding protein complexes as observed in the Protein Data Bank (PDB). Notable examples include p300 bound to nucleosome histone tails, SETD2 interaction with an H3 tail of partially unwrapped nucleosome, and FACT bound to subnucleosome.

(D) Rank of proteins with histone binding capabilities based on SF scores. The graphical line represents the normalized SF scores. The black lines indicate the proteins with known PDB structure interacting with nucleosomal histones. Binders interacting with partially unfolded nucleosomes or histone tails are colored in green.

Figure S2

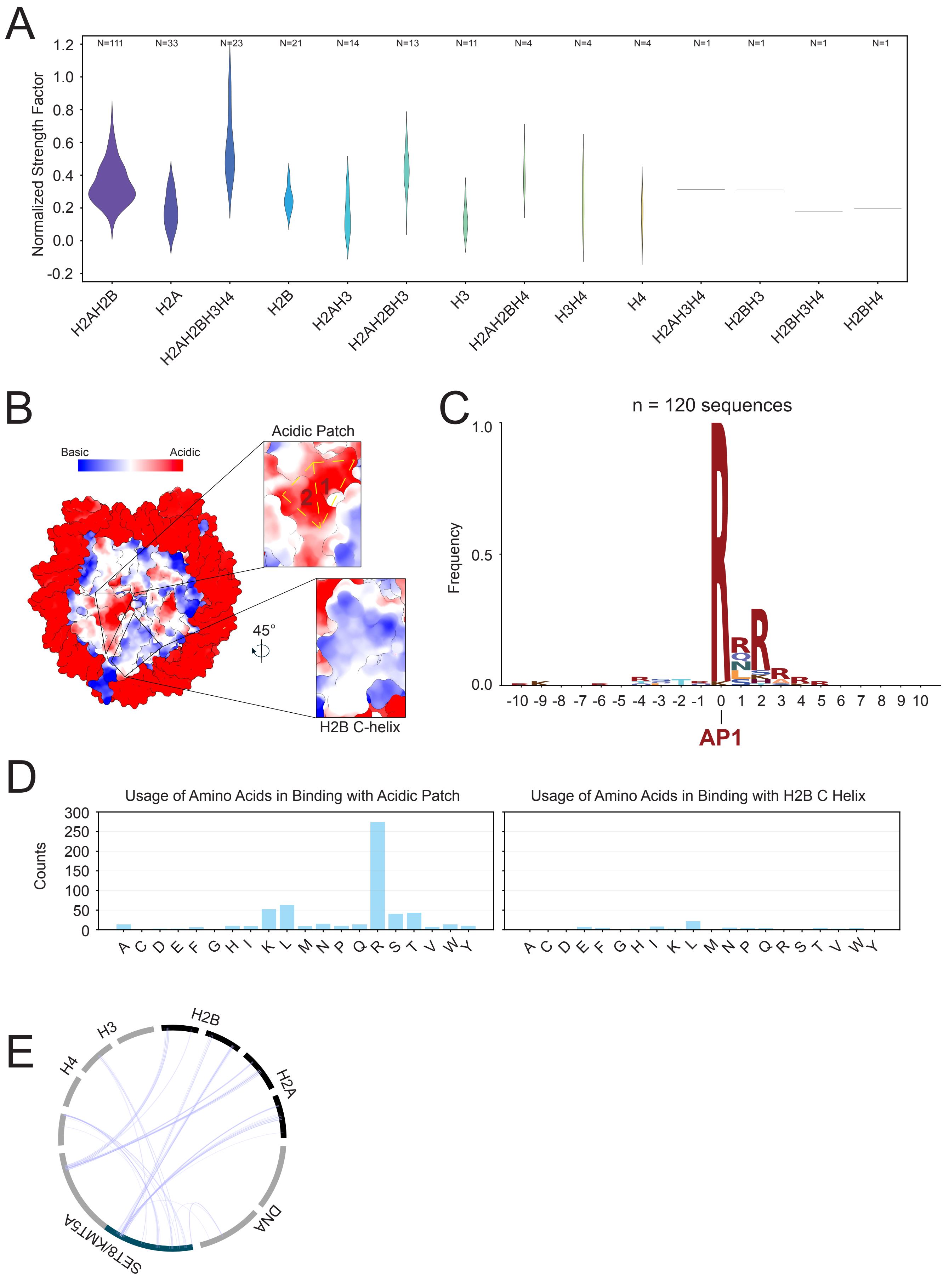
A
Normalized Strength Factor
N=111
N=33
N=23
N=21
N=14
N=13
N=11
N=4
N=4
N=4
N=1
N=1
N=1
N=1
H2AH2B
H2A
H2AH2BH3H4
H2B
H2AH3
H2AH2BH3
H3
H2AH2BH4
H3H4
H4
H2AH3H4
H2BH3
H2BH3H4
H2BH4
B
Basic
Acidic
Acidic Patch
45°
H2B C-helix
C
n = 120 sequences
Frequency
AP1
D
Usage of Amino Acids in Binding with Acidic Patch
Usage of Amino Acids in Binding with H2B C Helix
Counts
E
H2B
H3
H4
H2A
DNA
SET8/KMT5A

Figure S2. Comprehensive analysis of predictive nucleosome binders, related to Figure 1.

(A) Distribution of binding interfaces across various histone combinations, indicated by the width of each violin plot, showing the prevalence of specific histone interactions among nucleosome binders.

(B) Structural mapping of conserved H2A-H2B dimer interaction interfaces within the nucleosome. The acidic patch is subdivided into AP1 and AP2, while the entire H2B C-helix is shown. AP1 includes residues E62, D91, and E93, with AP2 comprising E62, D91, and E65.

(C) Sequence logo representation highlighting the predominance of arginine in the sequences near the acidic patch, analyzed from top 218 potential binders with a cutoff of PAE <= 10Å.

(D) Statistical analysis of amino acid involvement at two key interfaces: the acidic patch and the H2B C-helix, on the H2A-H2B complex.

(E) Visualization of amino acid pairs forming effective contacts in the predicted interaction between the SET8 and the nucleosome, illustrating specific binding sites and interactions.

Figure S3

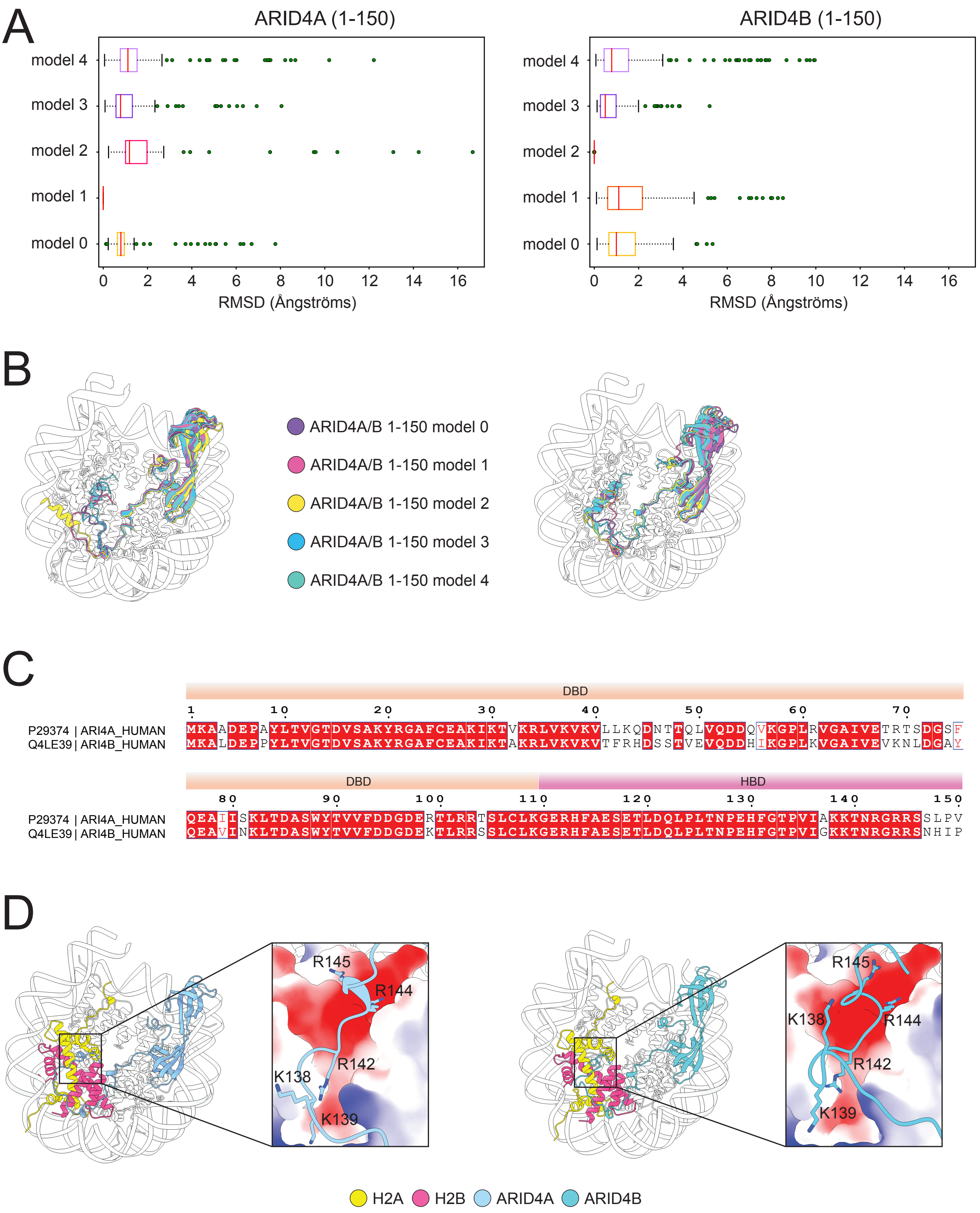

A
ARID4A (1-150)
ARID4B (1-150)
model 4
model 3
model 2
model 1
model 0
0 2 4 6 8 10 12 14 16
RMSD (Ångströms)
B
ARID4A/B 1-150 model 0
ARID4A/B 1-150 model 1
ARID4A/B 1-150 model 2
ARID4A/B 1-150 model 3
ARID4A/B 1-150 model 4
C
DBD
1 10 20 30 40 50 60 70
P29374 | ARI4A_HUMAN MKAADEPAYLTVGTDVSAKYRGAFCEAKIKTVKRLVKVKVLLKQDNTTQLVQDDQVKGPLRVGAIVETRTSDGSF
Q4LE39 | ARI4B_HUMAN MKALDEPPYLTVGTDVSAKYRGAFCEAKIKTAKRLVKVKVTFRHDSSTVEVQDDHIKGPLKVGAIVEVKNLDGAY
DBD
HBD
80 90 100 110 120 130 140 150
P29374 | ARI4A_HUMAN QEAIISKLTDASWYTVVFDDGDERTLRRTSLCLKGERHFAESETLDQLPLTNPEHFGTPVIAKKTNRGRRSSLPV
Q4LE39 | ARI4B_HUMAN QEAVINKLTDASWYTVVFDDGDEKTLRRSSLCLKGERHFAESETLDQLPLTNPEHFGTPVIGKKTNRGRRSNHIP
D
R145
R144
R142
K138
K139
H2A
H2B
ARID4A
ARID4B

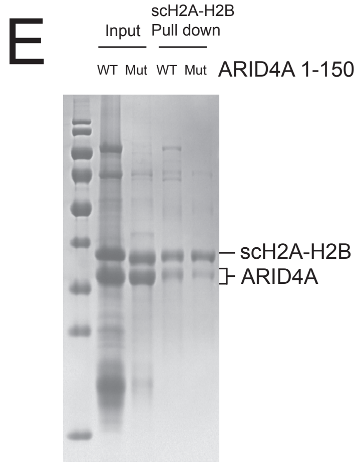

Figure S3. Comprehensive analysis of ARID4A and ARID4B, related to Figure 3.

(A) Root Mean Square Deviation (RMSD) statistics of ARID4A 1-150 (left) and ARID4B 1-150 (right) of all five structural models compared with the model with the higest SF score, in which each dot stands for RMSD of one amino acid in a single model.

(B) Superimposition of the different AlphaFold models of ARID4A/B 1-150, illustrating their structural consistency.

(C) Sequence alignment between ARID4A (1-150) and ARID4B (1-150). DBD: DNA binding domain, HBD: histone binding domain.

(D) Interaction details at acidic patch. Cartoon view shows ARID4A and ARID4B adopts a similar binding mode at acidic patch.

(E) Pulldown assay between histone and ARID4A 1-150. Single chain proteinA tagged H2A-H2B was immobilized on IgG beads as the bait, while ARID4A 1-150 or ARID4A 1-150 mutant was supplied as the prey.

Figure S4

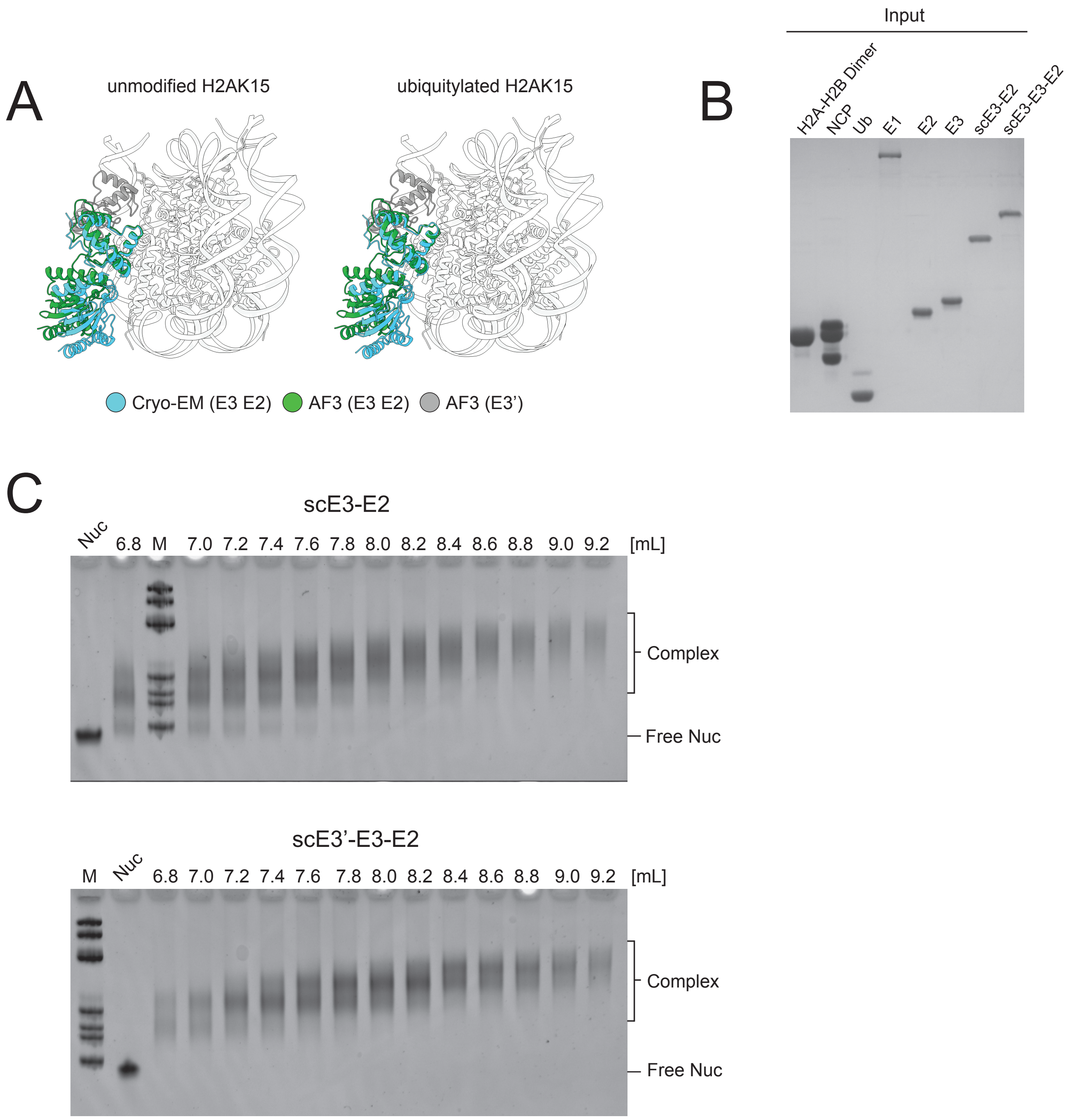

Figure S4. Characteristics of RNF168-nucleosome complex, related to Figure 4.
(A) Superposition between AlphaFold3 structures and cryo-EM structures. The left shows the human nucleosome core particle in complex with RNF168-UbcH5c (PDB: 8UPF) and the right shows the human nucleosome core particle ubiquitylated at histone H2A lysine 15 in complex with RNF168-UbcH5c (PDB: 8U14).
(B) SDS-PAGE of RNF168, UbcH5c and nucleosome core particle used in ubiquitination assay.
(C) Native-PAGE analysis of nucleosome-bound scE3-E2 and scE3'-E3-E2 subject to Gradient Fixation treatment. Top: scE3-E2; Bottom: scE3'-E3-E2. The scE3'-E3-E2 nucleosome complex shows reduced 'Free Nuc' band intensity, indicating stronger nucleosome engagement.

Figure S5

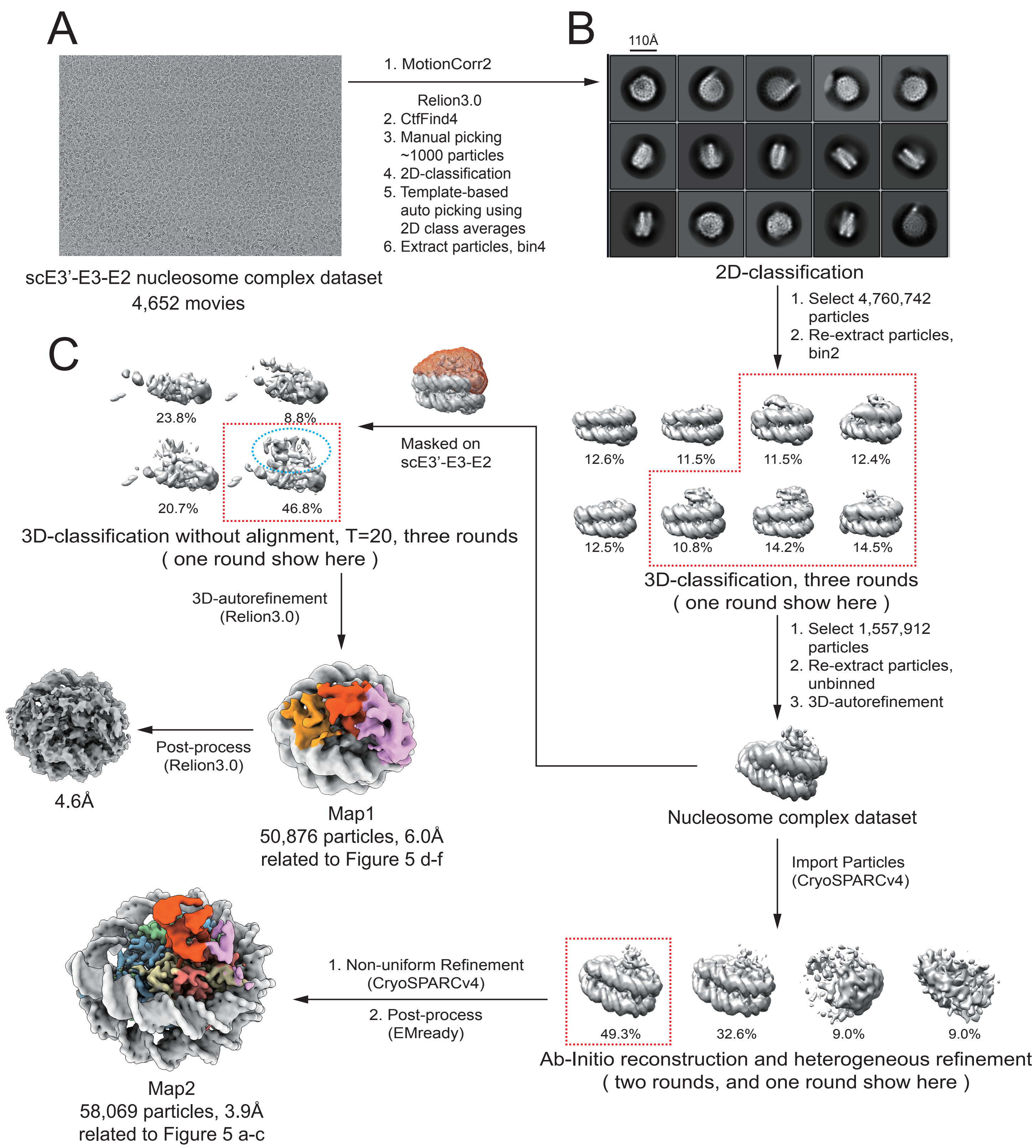

Figure S5. Cryo-EM data processing of sc-E3'-E3-E2 in complex of nucleosome, related to Figure 5.
(A) Representive cryo-EM micrograph of scE3'-E3-E2 in complex of nucleosome from 4652 movies.
(B) Selected 2D-classification of scE3'-E3-E2 complex with nucleosome. Scalebar is 11nm. Box size is 256, pixel size is 1.07 Å.
(C) Workflow of the image processing of 3D-classification, model reconstruction and refinement.

Figure S6

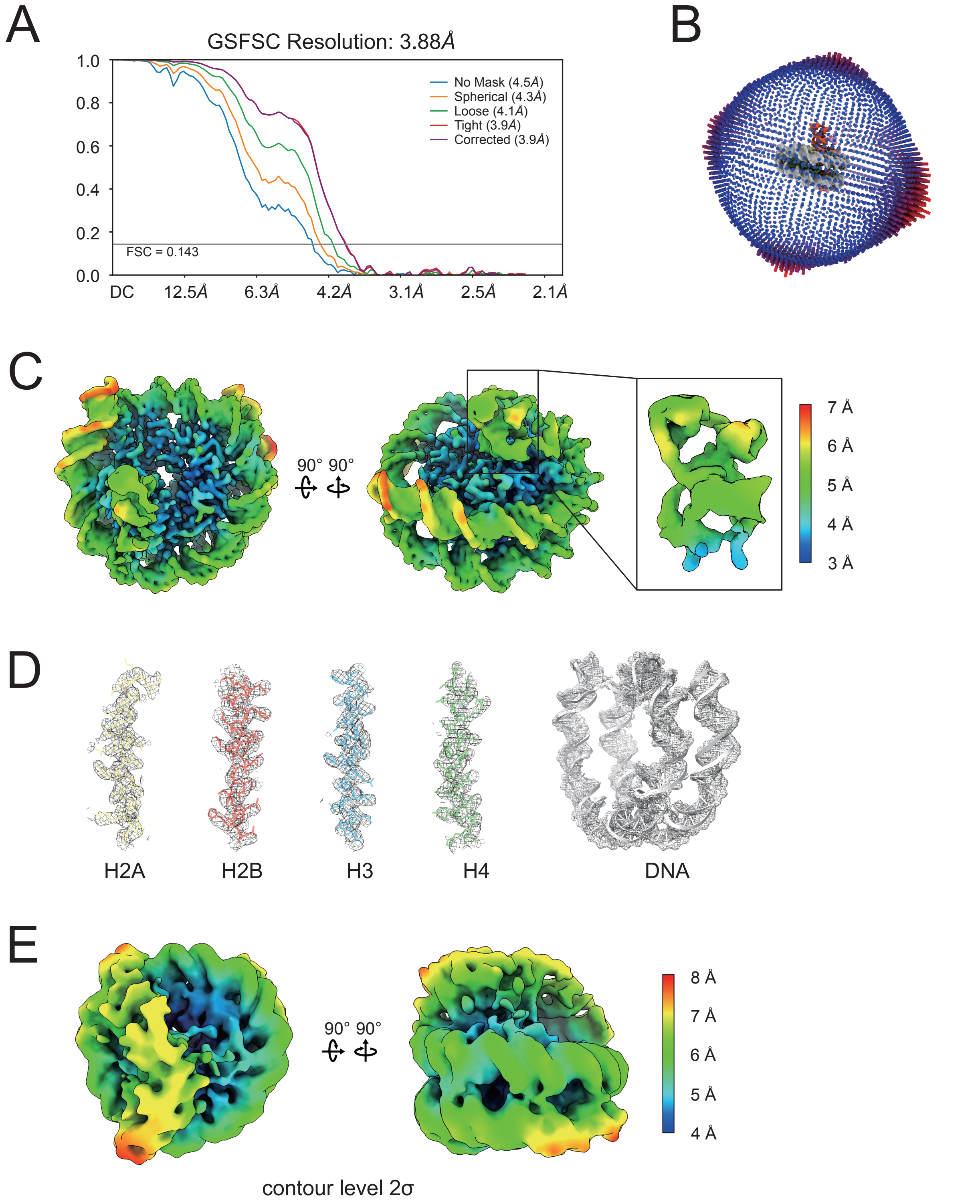

Figure S6. Structural analysis of sc-E3'-E3-E2 in complex of nucleosome, related to Figure 5.
(A) Fourier Shell Correlation (FSC) curve plot of scE3'-E3-E2 in complex of nucleosome with mask.
(B) Euler angle distribution of particles used in the final 3D reconstruction.
(C) Local-resolution map of scE3'-E3-E2 complex with nucleosome but no discernible density for E3' or E2.
(D) Cryo-EM density of histone (left) and DNA (right).
(E) Local-resolution map of scE3'-E3-E2 complex with nucleosome.

Figure S7

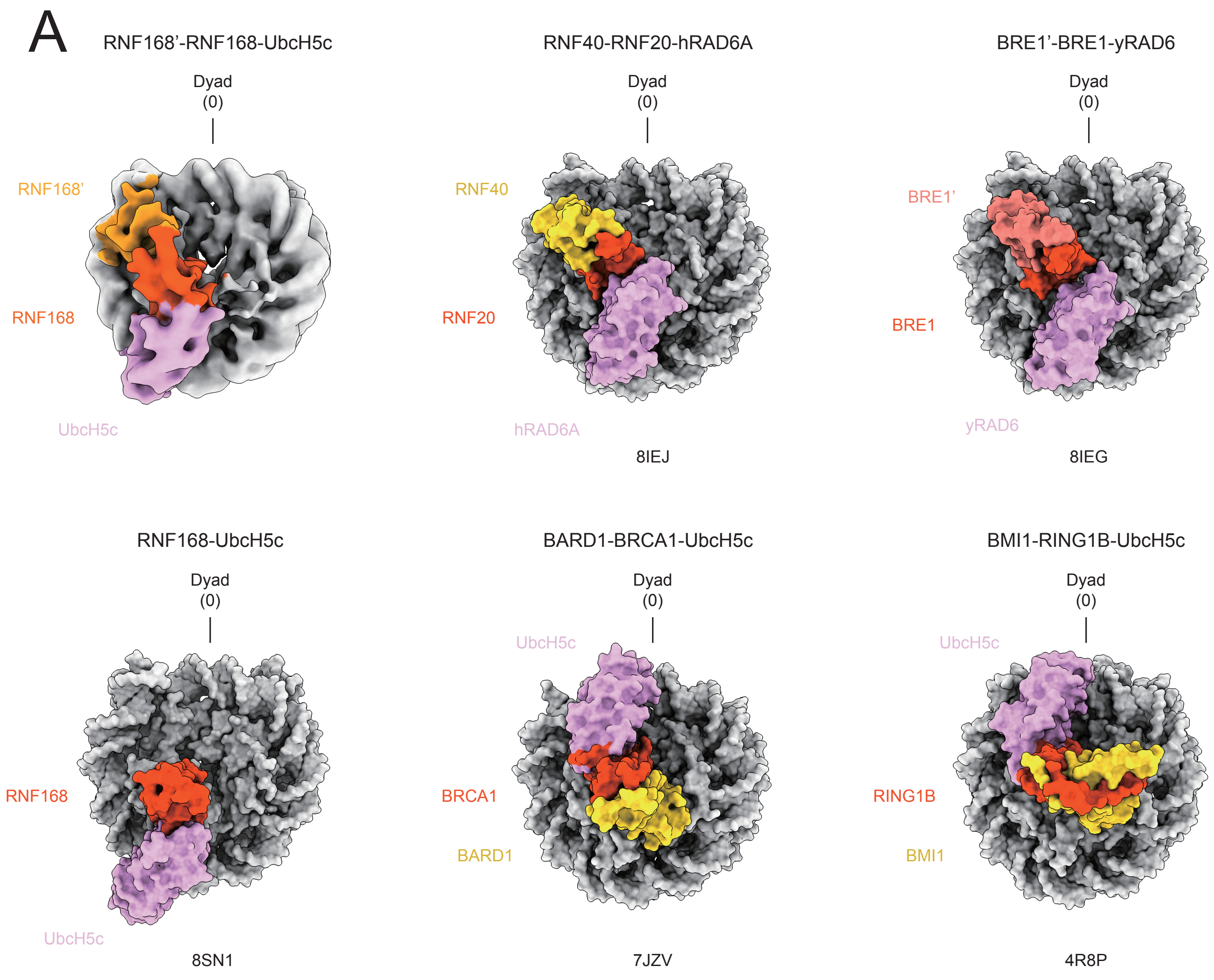

Figure S7. Comparison of RING-type ubiquitin ligases bound to nucleosome, related to Figure 5.

Table S1

Supplementary Table S1. Detailed list of human proteins linked to nucleosome complex structures in the Protein Data Bank (PDB)

Num	PDB ID	PDB release	Gene name	UniProt ID	Remark
1	8X7K	2024-08-07	RNF168	Q8IYW5	*
2	9FH9	2024-07-24	CCNB1	P14635	*
3	8U5H	2024-06-12	DNMT3A	Q9Y6K1	*
4	8PP6	2024-04-03	RYBP	Q8N488	*
5	8X1C	2024-03-06	VPS72	Q15906	*
6	7YRG	2023-12-13	KMT5B	Q4FZB7	*
7	8UX1	2023-11-22	RCC1	P18754	*
8	8HQY	2023-09-27	SSX2	Q16385	*
9	8HR1	2023-09-13	SSX1	Q16384	*
10	8IEJ	2023-09-06	BRE1A	Q5VTR2	*
11	7UV9	2023-02-22	KDM2A	Q9Y2K7	*
12	8H1T	2023-02-01	BAP1	Q92560	*
13	7Y8R	2022-12-07	SNF5	Q12824	*
14	7XPX	2022-06-01	KMT5A	Q9NQR1	*
15	7TAN	2022-05-04	VRK1	Q99986	*
16	7LYC	2021-06-16	BARD1	Q99728	*
17	6ZHX	2020-12-23	CHD1L	Q86WJ1	*
18	6Y5E	2020-09-23	CGAS	Q8N884	*
19	6SE6	2019-08-14	CENPC	Q03188	*
20	5KGF	2016-07-27	TP53BP1	Q12888	*
21	8PP7	2024-03-27	RING2	Q99496	*
22	8OF4	2023-08-09	SIR6	Q8N6T7	*
23	7LYB	2021-07-28	BRCA1	P38398	*
24	8VG1	2024-08-07	GATA4	P43694	-
25	9FGQ	2024-07-24	CDC27	P30260	-
26	8X19	2024-03-06	ZNHI1	O43257	-
27	8GUJ	2023-09-20	BRE1B	O75150	-
28	7VDV	2022-05-18	SMCA4	P51532	-
29	7XCR	2022-04-20	DOT1L	Q8TEK3	-
30	6PA7	2020-06-17	DNMT3B	Q9UBC3	-
31	8X19	2024-03-06	DMAP1	Q9NPF5	*
32	8GPN	2023-02-15	MEN1	O00255	*
33	8DU4	2022-09-21	RBBP5	Q15291	*
34	7U46	2022-03-16	CENPN	Q96H22	*
35	6RYR	2020-07-15	CHD4	Q14839	*
36	6VYP	2020-05-27	RCOR1	Q9UKL0	*
37	6KIU	2019-09-11	KMT2A	Q03164	*
38	6R8Y	2019-06-12	DDB2	Q92466	*
39	8PKI	2024-02-28	NR5A2	O00482	-
40	8G6G	2024-02-21	UBB	P0CG47	-
41	8SMY	2024-01-17	UBE2D3	P61077	-
42	8WG5	2023-12-27	USP16	Q9Y5T5	-
43	8IEJ	2023-09-06	UBE2A	P49459	-
44	8H1T	2023-02-01	ASXL1	Q8IXJ9	-

Supplementary Table S1. Detailed list of human proteins linked to nucleosome complex structures in the Protein Data Bank (PDB)

Num	PDB ID	PDB release	Gene name	UniProt ID	Remark
45	7SCY	2022-01-12	PARP1	P09874	-
46	7BWD	2021-04-14	RPS27A	P62979	-
47	7K6Q	2021-02-10	UBC	P0CG48	-
48	6KIW	2019-09-11	KMT2C	Q8NEZ4	-
49	8JNE	2024-03-27	RAD51	Q06609	-
50	8PP7	2024-03-27	BMI1	P35226	-
51	8UX1	2023-11-22	RAN	P62826	-
52	8BYQ	2023-05-03	GTF2H5	Q6ZYL4	-
53	8BYQ	2023-05-03	ERCC3	P19447	-
54	7ZI4	2023-04-19	INO80C	Q6PI98	-
55	7VDV	2022-05-18	ARID2	Q68CP9	-
56	8YV8	2024-07-31	CDCA7	Q9BWT1	subNuc
57	8OX1	2023-08-30	TERF1	P54274	subNuc
58	7Y60	2023-08-16	CHAF1B	Q13112	subNuc
59	8OTS	2023-05-24	MYC	P01106	subNuc
60	8OTS	2023-05-24	MAX	P61244	subNuc
61	6T7B	2020-04-29	SOX2	P48431	subNuc
62	6T7C	2020-04-29	SOX11	P35716	subNuc
63	6UPL	2019-12-11	SSRP1	Q08945	subNuc
64	6UPL	2019-12-11	SUPT16H	Q9Y5B9	subNuc
65	8V4Y	2024-03-20	SMARCA5	O60264	tail
66	8X1C	2024-03-06	ACTR6	Q9GZN1	tail
67	8CBQ	2023-09-06	PSIP1	O75475	tail
68	7XX6	2023-05-31	H1-0	P07305	tail
69	8HAI	2023-05-17	EP300	Q09472	tail
70	8HAL	2023-05-17	CREBBP	Q92793	tail
71	7Y8R	2022-12-07	PHF10	Q8WUB8	tail
72	7EA8	2021-07-14	SETD2	Q9BYW2	tail
73	7CRR	2020-10-21	NSD3	Q9BZ95	tail
74	7CRO	2020-10-21	NSD2	O96028	tail
75	6R1U	2019-04-24	KDM1B	Q8NB78	tail

Proteins 1-30 are identified as nucleosome acidic patch binders, and proteins 1-55 as histone binders. Proteins accurately predicted are marked with a star (*), while those inaccurately predicted are noted with a minus (-). Proteins 56-75, classified as either subnucleosome binders (subNuc) or histone tail binders (tail), are excluded from this analysis.

Table S2

Supplementary Table S2. Construction of RING-family ubiquitin E3 ligases in AF3

Abbr.	This study (human)	human BRCA1	yeast BRE1A	human RING1B
E3' E3 E2	RNF168 1-189<br>RNF168 1-189<br>UbcH5c 1-147	BRCA1 4-97<br>BARD1 32-118<br>UbcH5c 2-147	BRE1 637-700<br>BAE1 637-700<br>RAD6 3-150	RING1B 15-115<br>BMI1 3-107<br>UbcH5b 2-147
E3 E2	RNF168 1-103<br>UbcH5c 1-147	BRCA1 4-97<br>UbcH5c 2-147	BRE1 637-700<br>RAD6 3-150	RING1B 15-115<br>UbcH5b 2-147
E3	RNF168 1-103	BRCA1 4-97	BRE1 915-974	RING1B 15-115
E2	UbcH5c 1-147	UbcH5c 2-147	RAD6 3-150	UbcH5b 2-147
PDB (Source)		7LYB	8IEG	4R8P, 8GRM

Table S3

Supplementary Table S3. Cryo-EM data collection, refinement, and validation statistics

Data collection	scRNF168'-RNF168-UbcH5c and NCP complex (Map2)
PDB entry	9KQ2
EMDB entry	EMD-62494
Magnification	130,000
Voltage (kV)	300
Detector	K3
Electron exposure (e-/Å2)	50
Defocus range (μm)	-1.8 to -2.2
Pixel size (Å)	1.07
Symmetry imposed	C1
Micrographs (no.)	4652
Final particles (no.)	58069
Map resolution (Å)	3.9
FSC threshold	0.143
Model Building and Refinement	
Software	ChimeraX
Refinement	Phenix
Initial model used (PDB code)	4GB0, 3LZ0
Chains	12
Atoms	12572
Residues	Protein: 834 Nucleotide: 290
Ligands	Zn^{2+}: 2
Validation	
MolProbity score	1.69
Clashscore	8.25
Rotamer outliers (%)	1.28
Bond lengths (Å)	0.005 (0)
Angles (°)	0.719
Ramachandran plot	
Outliers	0.00
Allowed	2.94
Favored	97.06
